# Increased Endothelial Inflammation, sTie-2 and Arginase Activity in Umbilical Cords Obtained from Gestational Diabetic Mothers

**DOI:** 10.1371/journal.pone.0084546

**Published:** 2013-12-20

**Authors:** Hemant Giri, Shivam Chandel, Linga S. Dwarakanath, Sooriyakala Sreekumar, Madhulika Dixit

**Affiliations:** 1 Laboratory of Vascular Biology, Department of Biotechnology, Indian Institute of Technology Madras, Chennai, India; 2 Sooriya Hospital, Chennai, India; UAE University, Faculty of Medicine & Health Sciences, United Arab Emirates

## Abstract

**Objective:**

The aim of this study was to determine subclinical inflammation in umbilical vein derived endothelial cells (HUVECs) obtained from Asian Indian subjects with gestational diabetes (GDM) and to determine levels of angiogenic factors and arginase activity in their cord blood.

**Methods:**

This case-control study included 38 control and 30 GDM subjects. Subjects were confirmed as GDM based on 75g oral glucose tolerance test (OGTT) conducted in the second trimester of pregnancy. Angiogenic markers and arginase activity were measured in cord blood by ELISA and colorimetric methods respectively. Endothelial inflammation was assessed through adhesion of PKH26-labelled leukocytes onto HUVEC monolayer obtained from the study groups. Gene and surface expression of adhesion molecules were confirmed via reverse transcription polymerase chain reaction (RT-PCR) and flow cytometry respectively.

**Results:**

The study revealed increased adhesion of leukocytes to HUVECs isolated from GDM subjects compared to controls. HUVECs of babies born to GDM mothers had increased surface and mRNA expression of E-selectin. sTie2 levels were significantly higher in the cord blood for GDM subjects (3869 ± 370 ng/L) compared to controls (3045 ± 296 ng/L). Furthermore, arginase activity was higher in cord blood of GDM mothers as opposed to the control group (7.75 ± 2.4 µmoles of urea/ml/hour vs 2.88 ±0.49 µmoles of urea/ml/hour; p-value= 0.019). Spearman’s correlation analysis revealed positive correlation of cord blood arginase activity with glucose intolerance (ρ=0.596, p=0.004) and post load glucose values (ρ=0.472, p=0.031) of mothers observed during the second trimester of pregnancy.

**Conclusions:**

HUVECs derived from Asian Indian GDM mothers, exhibit signs of sub-clinical endothelial inflammation along with increased levels of sTie2 and arginase activity in their cord blood serum.

## Introduction

Angiopoietins and vascular endothelial growth factor (VEGF) play major roles in placental growth and embryonic angiogenesis [1,2]. For instance, angiopoietin-1 (Ang-1) via Tie2 signalling mediates endothelial survival, stabilization and anti-inflammatory functions. In contrast, angiopoietin-2 (Ang-2) acts as an Ang-1 antagonist by binding to the Tie2 receptor and thus induces vascular leakage, inflammation and cancer metastasis [3]. Ang-2 also sensitizes endothelium towards pro-inflammatory effects of sub-optimal concentrations of TNF-α [4]. Circulating levels of Ang-2 is thus a promising marker of early cardiovascular diseases and endothelial inflammation [5,6]. Interestingly, with increasing gestational age, the Ang-1 levels increase in the amniotic fluid while those of Ang-2 decreases in normal pregnancy. In contrast, for women with intra-amniotic inflammation, levels of Ang-2 and sTie2 are increased in the amniotic fluid [7]. Recently, serum levels of angiopoietin related growth factor (AGF) were shown to be elevated during the third trimester in gestational diabetes [8] however, not much is known about the levels of angiopoietins in cord blood of healthy and gestational diabetes mellitus subjects (GDM). 

Gestational diabetes is a major risk for the mother and the foetus at the time of pregnancy and contributes to the increasing epidemic of diabetes [9]. It increases the risk for developing overt diabetes in mothers at later stages of life [10]. These women also have increased circulating levels of pro-inflammatory markers TNF-α, hsCRP and PAI-1, all of which inversely correlate with endothelial function [11,12]. Additionally, the intra-uterine environment consisting of hyperglycemia, hyperinsulinemia and insulin resistance are associated with endothelial dysfunction in their offspring [13]. These factors possibly increase the permeability of the foeto-placental circulation and thus lead to transmission of inflammatory mediators from the mother to the foetal circulation. Although increased carotid intima media thickness (IMT) is reported in GDM women [14,15], endothelial inflammation an early event in atherosclerosis, is not well characterized in them and foetal circulation. Given that prevalence of gestational diabetes in Asian women is 2-3 folds higher than Caucasians [16-18], we sought to determine the status of endothelial inflammation in human umbilical vein derived endothelial cells (HUVECs) obtained from babies born to Asian Indian GDM women. 

Nitric oxide (NO) prevents inflammation [19] and its bioavailability is regulated through an interplay between major NO synthesizing enzyme, endothelial nitric oxide synthase (eNOS), and its competitive inhibitor arginases [20]. Arginases also compromise pregnancy-associated immune-tolerance as consequent L-arginine depletion leads to impaired lymphocyte responses [21]. Recent studies with arginase-II knock-out mice and cell culture systems have highlighted their significance in inflammation, atherosclerosis and glucose intolerance [22]. However, involvement of arginases, if any, in GDM is presently elusive. Thus, the other aim of the study was to determine arginase activity and angiogenic factors in the cord blood of infants born to GDM subjects. 

## Materials and Methods

MCDB 131 Basal Medium from Himedia Labs, Mumbai, India along with growth supplements was used to culture endothelial cells isolated from umbilical cords. Foetal Bovine serum of South American origin was from GIBCO, Invitrogen, NY, USA. Antibodies for flow cytometry and fibronectin were from Beckton Dickinson, USA. Arginase inhibitor (BEC hydrochloride) was from Calbiochem, Inc. La Jolla, CA. M-MuLV reverse transcriptase and DNA polymerase enzymes were from New England Biolabs, UK. PCR primers, L-Arginine, histopaque solution, PKH26, and all the other lab chemicals were from Sigma Aldrich, St. Louis, MO unless specified otherwise.

### Study subjects

Experimental procedures involving human tissue samples and blood were reviewed and approved by the IIT Madras institutional ethics committee in accordance with Declaration of Helsinki [IITM-IEC No:2009022]. Informed written consent was obtained from the study subjects and both the study and the consent forms were approved by the institutional ethics committee. Umbilical cord and cord blood from study subjects were collected from Sooriya hospital, Chennai, India. A total of 68 subjects; 38 subjects in the control group and 30 subjects in the GDM group were recruited. Subjects were classified as GDM based on fasting plasma glucose values ≥ 5.1mmol/l) [23] and post load plasma glucose values at 2 hour ≥7.8mmol/l for 75g OGTT performed during the second trimester of pregnancy according to Diabetes in Pregnancy Study group India (DIPSI) guidelines [24]. For glucose management, all GDM subjects were on diet and exercise except for two who were on insulin. Following exclusion criteria were applied: hypertension, diabetes, abortion, foetal death, cigarette smoking, ongoing medication, family history of hypertension and presence of obesity, defined as, mother’s weight >90 Kg at the time of booking as per NICE clinical guidelines for management of diabetes and its complications [25]. For all experiments involving adhesion assays, FACS analysis or RT-PCR, a minimum of 9 subjects was taken from each group. PBMCs were isolated using ficoll density gradient as described earlier [26] and following labelling with PKH-26 as per manufacturer’s instructions, labelled leukocytes were used for adhesion assay. 

### Human Umbilical Vein Endothelial Cell (HUVEC) isolation and culture

Endothelial cells from respective cords were isolated for culture within 3 hours of birth as described earlier [27]. Briefly, freshly obtained cords were gently squeezed to collect the cord blood, followed by gentle washing with sterile phosphate buffer saline (PBS). Endothelial cells were isolated with collagenase digestion and the cells were cultured on fibronectin coated tissue culture dishes from Corning. Cells were cultured in MCDB 131 growth medium until they reached confluence.

### Adhesion assay

Confluent cultures of HUVECs (passage P_0_) from the two study groups were serum starved for 6-8 hours prior to adhesion assay. Labelled leukocytes were incubated with HUVECs at 37°C and 5% CO_2_ for 3 hours. Cells were then washed with PBS thrice to remove non-adherent leukocytes. Adhesion was assessed as the number of leukocytes adhered to HUVEC monolayer per field view. Each experiment was performed in triplicates and data are represented as mean±SEM.

### RTPCR

Total RNA was isolated from 5 X 10^6^ cultured endothelial cells (passage P_0_) from study subjects using TRIZOL reagent (from Sigma) following manufacturer’s instructions. Following DNAse treatment, total RNA (1μg) was reverse transcribed (RT) using M-MuLV RT enzyme and Oligo dT_12-18_ primers. A reaction performed in absence of reverse transcriptase enzyme acted as negative control. Amplified cDNA was subjected to PCR using gene specific primers as listed in Table S1. RT-PCR was also performed for GADPH as internal control.

### Cord blood collection and ELISA

Cord blood was collected by gently squeezing the cord and hence it was pooled from both the arteries and the vein of the umbilical cord. Collected blood was allowed to clot at room temperature. Serum was separated by centrifugation at 4000rpm for 10 minutes, and aliquots of serum were stored at −80°C until further analyses. Serum Ang-1, Ang-2, Tie-2 and VEGF concentrations were determined in duplicate by ELISA according to the manufacturer’s instructions (R&D Systems, Inc., Minneapolis, MN). 

### Arginase activity

Arginase activity was measured according to the method described earlier [28]. 100 µl of serum was incubated with equal volumes of 10 mM manganese chloride in 50 mM Tris-HCl, pH 7.4 at 55°C for 10 minutes to activate the enzyme. Activated extracts were incubated with 0.1M L-arginine (pH 9.7) in presence or absence of 200µM BEC (Calbiochem), a competitive inhibitor of arginase. The assay mixtures were incubated at 37°C in a shaking water bath for two hours. The reaction was stopped by adding 400 µl of the acid mix consisting of sulphuric acid, phosphoric acid and water in a ratio of 1:3:7. 25 µl of 9% ISPF (α-isonitrosopropiophenone) dissolved in ethanol was added and tubes were incubated in boiling water bath for 45 minutes. The tubes were cooled in dark at room temperature for 10 minutes and aliquots of 200 µl were transferred to a 96-well plate for absorbance at 540 nm. 

### Flow cytometry

Flow cytometric analysis was done as described earlier [29]. Confluent endothelial cells were washed with ice cold 1X PBS and then fixed with 0.5% paraformaldehyde for 5-10 minutes. Cells were then washed with 1X PBS and blocked with 0.1% BSA. Finally, anti-ICAM-1 PE, anti-E-selectin PE and Isotype PE antibodies were added and incubated at 4°C overnight. Cells were washed twice with 1X PBS, trypsinized and resuspended in 250μl of 1X PBS for analysis. A minimum of 10,000 cells were scored for surface expression of adhesion molecules in FACS Canto (Beckton Dickinson, USA). The FACS operator was blinded to the clinical status of the subjects and the data was analyzed using Flowjo software program (Version 7.6.1).

### Statistics

Clinical data are expressed as mean ± SEM. Kolmogorov-Smirnov test was used to determine normality. Parametric variables were analysed by two tailed student’s t-test, while non parametric data were analyzed by Mann Whitney test. Correlations between serum concentrations of Ang-1, Ang-2 and VEGF with arginase activity were analyzed by Spearman’s rank correlation. 

## Results

Anthropometric and biochemical parameters for gestational diabetes and control group are summarized in table 1. Except for four, all the other subjects delivered vaginally. Among the recruited subjects 9 in control and 15 in GDM group, had family history of diabetes. Fasting and post-load blood glucose values were significantly high in gestational diabetes group as compared to the control group. There were no significant differences in age, blood pressure and child weight between the two groups. Health of the newborns were assessed with Apgar score at 1min and 5 min after birth, and all were found to be healthy in both the groups with minimum 6 points on Apgar score in accordance with established guidelines [30].

**Table 1:** Anthropometric and biochemical characteristics of study subjects.

**Anthropometric measures**	**Control (N=38)**	**GDM (N=30)**
Age (Years)	27.18 ± 0.53	27.32 ± 0.54
Weight (Kg) (12-13 weeks)	66.38 ±2.28	63.25 ±1.93
Systolic Blood Pressure (mmHg) (II trimester)	118.7 ± 0.55	118.0 ± 1.00
Diastolic Blood Pressure (mmHg) (II trimester)	81.37 ± 2.70	77.0 ± 0.85
Systolic Blood Pressure (mmHg) (III trimester)	120 ± 0.37	119.7 ± 0.58
Diastolic Blood Pressure (mmHg) (III trimester)	78.63 ± 0.55	79.0 ± 0.73
Weight of child (Kg)	3.0 ± 0.05	3.11 ± 0.05
**Biochemical measures**		
Fasting Glucose (mmol/L)	4.816 ± 0.05	5.483 ± 0.10^****^
Post load Glucose (mmol/L)	5.947 ± 0.17	8.517 ± 0.42^****^

P < 0.0001 versus controls.

### Increased endothelial inflammation

**Figure 1:**
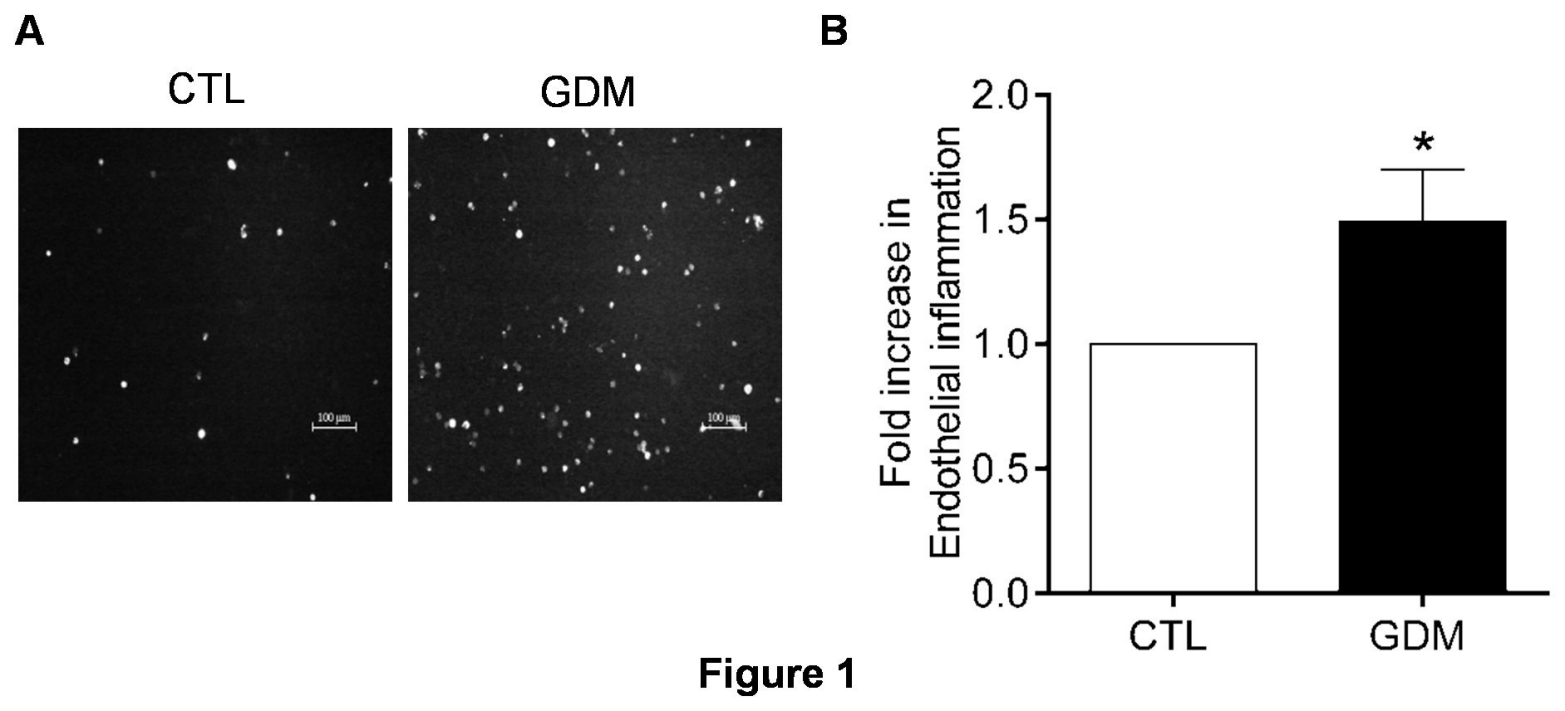
Subclinical inflammation in HUVECs isolated from GDM women. (A) Representative picture depicting adherence of PKH26 labelled leukocytes to human umbilical vein–derived endothelial cell (HUVEC) monolayer of control and GDM subjects. (B) Bar graph summarizing fold increase in adhesion of leukocytes to HUVECs for a minimum of 10 subjects in each group. Inflammation was scored as number of leukocytes adhered per field view. *p≤0.05 represents statistical significance versus control.

**Figure 2:**
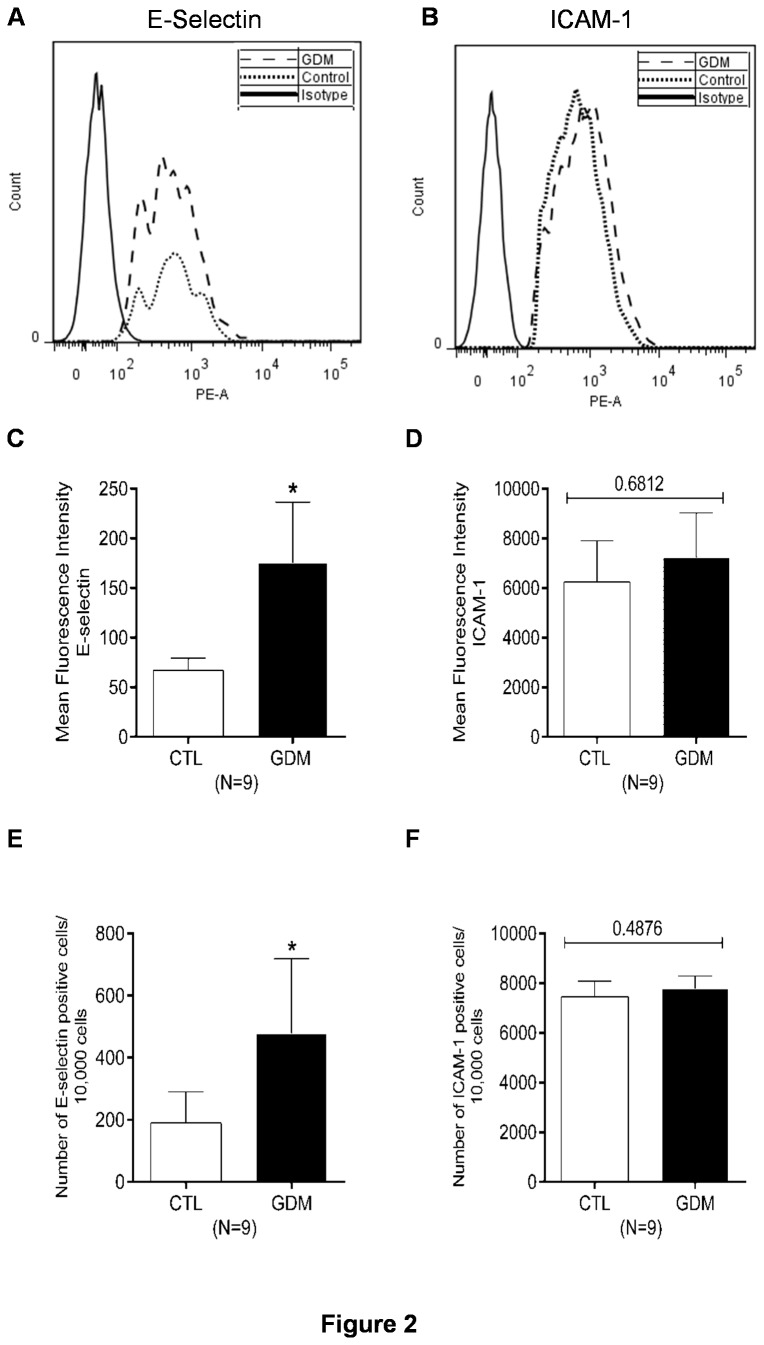
FACS analysis for surface expression of adhesion molecules. (A&B) Histograms depicting mean fluorescence intensity for surface expression of E-selectin and ICAM-1 in isolated HUVECs (P_0_) from control and GDM women, (C&D) Bar graphs summarizing MFI for E-selectin and ICAM-1 for nine subjects in each group, (E&F) Bar graphs summarizing number of cells positive for E-selectin and ICAM-1 in freshly isolated HUVECs. *p≤0.05 versus control group.

**Figure 3:**
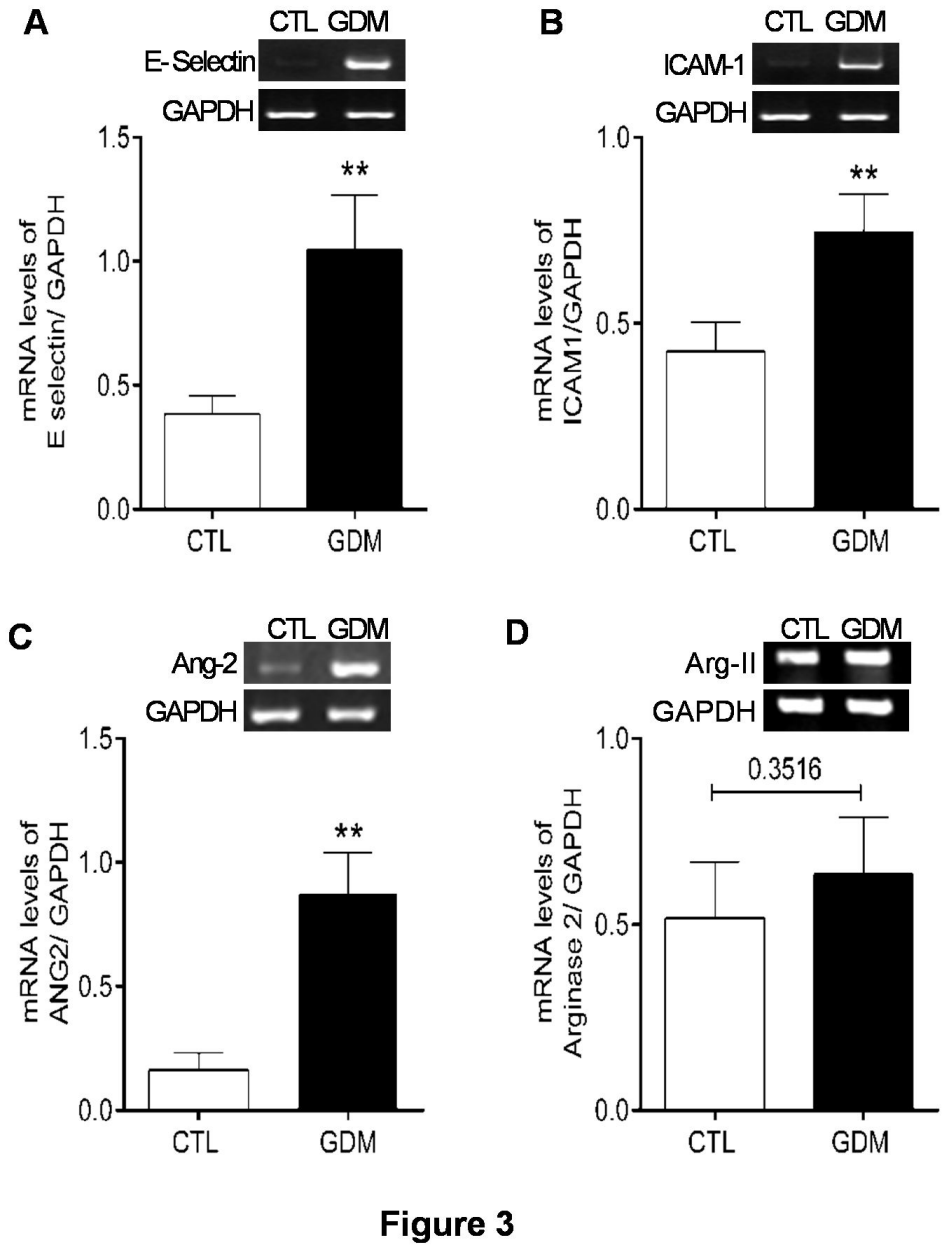
mRNA expressions profile of E-selectin, ICAM-1, Ang-2 and Arginase II in HUVECs. Increased mRNA expression of E-selectin (A), ICAM-1 (B), Ang-2 (C) and arginase II (D) with their representative gel pictures and cumulative bar graphs. RT-PCR analyses were done for 12 controls and 15 GDM subjects. **p≤0.01 versus control group.

Subclinical inflammation in women with history of gestational diabetes is already reported [31,32]. However, not much is studied immediately after delivery. The circulating levels of adhesion molecules like vascular cell adhesion molecule-1 (VCAM-1), intercellular adhesion molecule-1 (ICAM-1) and E-selectin are also associated with inflammation and cardiovascular diseases [33,34]. However, nothing is known about the status of endothelial inflammation in HUVECs derived from Asian Indian women. We hence isolated HUVECs from healthy and GDM mothers post delivery and performed adhesion assay with PKH26 labelled PBMC’s isolated from healthy subjects to estimate the status of endothelial inflammation in foeto-placental vasculature. HUVECs from newborns having gestational diabetic mothers, showed increased adhesion of freshly isolated leukocytes to endothelial monolayer in un-stimulated condition (Figure 1A&B). We also observed increased surface expression and number of E-selectin positive cells by FACS in HUVECs freshly isolated from cords of infants born to GDM mothers (Figure 2A, C&E). Expression of E-selectin mRNA in isolated HUVECs was also increased for GDM group (Figure 3A). However, no changes for surface expression of ICAM-1 were observed (Figure 2B, D&F) although increased mRNA expression for ICAM-1 was observed via RTPCR (Figure 3B) in HUVECs of GDM subjects. Expression of VCAM-1 mRNA remained unaltered between the two groups (not shown). 

### Cord blood analysis

To further assess the influence of gestational diabetes on circulating levels of angiopoietins in foetal circulation, we measured soluble Ang-1, VEGF, Ang-2, sTie2 and arginase activity in cord blood serum. As seen in Table 2, levels of sTie2 were increased for GDM samples, while levels of Ang-1, Ang-2 and VEGF were not different between the two groups. However, we found increased expression of Ang-2 mRNA in HUVECs isolated from these GDM cords (Figure 3C). 

**Table 2:** Cord blood serum levels of different angiogenic factors and arginase activity.

**Parameters**	**Control (N=24)**	**GDM (N=13)**	**P- value**
Angiopoietin 1 (ng/L)	6495 ± 179	6796 ± 132	0.2145
Angiopoietin 2 (ng/L)	1972 ± 262	1925 ± 333	0.9111
Arginase Activity (µmoles of urea/ml/hour)	2.88 ±0.49	7.75 ± 2.4*	0.0151
Tie 2 (ng/L)	3045 ± 296	3869 ± 370*	0.0464
VEGF (ng/L)	329 ± 61	287 ± 49	0.6178

^*^ P < 0.05 versus controls.

A recent report suggested that NO metabolism in circulation is mainly regulated by eNOS, rather than other isoforms of NOS [35]. Although, increased expression of eNOS is reported in HUVECs from GDM cords [13], the resulting NO levels in cord blood serum are unaltered [36]. This prompted us to determine arginase activity in cord blood of recruited subjects. We found increased arginase activity in the cord serum of babies born to GDM mothers as seen in table 2. We then performed Spearman’s correlation analysis of arginase activity as dependent variable with other cord blood parameters and plasma glucose values of mothers as independent variable. As seen in table 3, arginase activity exhibited a significant positive correlation with glucose intolerance and post load glucose values of mothers in second trimester. Among the angiogenic factors, Pearson’s correlation analysis demonstrated a negative correlation of Ang-1 with surface (r= -0.638, p=0.026) and gene expression (r= -0.607, p=0.036) of E-selectin.

**Table 3:** Spearman's correlation analysis of Arginase activity with other parameters.

**Parameters**	**Correlation coefficient (*ρ*)**	**p value**
Glucose Intolerance^†^	0.596	0.004
Fasting Glucose	0.349	0.121
Post load Glucose	0.472	0.031
**Cord Blood Parameters**		
Angiopoietin 1	0.296	0.192
Angiopoietin 2	0.244	0.287
Tie 2	0.308	0.175
VEGF	0.100	0.666

^†^ Serum glucose values ≥7.8mmol/l at 2 hours of 75g OGTT were considered as impaired glucose tolerance according to Diabetes in Pregnancy Study group India (DIPSI) guidelines [24]. For Correlation analysis, presence and absence of glucose intolerance was assigned the binary numerical system of 1 and 0 respectively.

## Discussion

Gestational diabetes imposes a great challenge for gynaecologists due to increased risk of the mother and the child developing overt diabetes and associated vascular complications [10,37,38]. For instance, GDM mothers exhibit higher mean arterial pressure and increased carotid intima-media thickness (IMT) [15] in addition to increased circulating levels of pro-atherogenic markers such as TNF-α, hsCRP, IL-6 and PAI-1 [11,12]. Among these, IMT is positively correlated with glucose intolerance and hypertension [15]. In the current study we observed increased adherence of peripheral blood derived leukocytes to HUVECs derived from Asian Indian GDM cords even in absence of pro-inflammatory cytokines in the culture medium, thereby reflecting enhanced endothelial inflammation in foetal vasculature. This was accompanied by a parallel increase in mRNA and surface expression of E-selectin on HUVECs derived from GDM subjects. This observation is consistent with an earlier finding reporting association of increased circulating levels of soluble E-selectin with early cardiovascular risk in GDM women [39]. It should be noted that endothelial inflammation triggers initiation and progression of atherosclerosis. To the best of our knowledge, we report for the first time increased adherence of leukocytes to HUVECs isolated from Asian Indian gestational diabetic women. 

Presence of high glucose in GDM subjects may be involved in endothelial inflammation, as there are reports of association of high maternal glucose with complications like Chorioamnionitis, an inflammation of the foetal membranes [40,41]. Obesity is one of the major causes of gestational diabetes and endothelial inflammation [42]. However, in the current study all recruited subjects were non-obese as the maternal weights were way below the cut off mark of 90 kg which is used as a screening parameter for determining obesity in pregnancy as per guidelines of NICE,UK and report on European study [43]. Maternal weight >90kg also results in development of macrosomia in foetus however, we did not observe macrosomia in newborns born to GDM mothers in the current study [44].

We observed a significant increase for sTie2 levels and arginase activity in cord blood serum of GDM women, while the levels of angiogenic factors (Ang-1, Ang-2 and VEGF) were unaltered. Among the angiopoietins, Ang-1 negatively correlated with gene and surface expression of E-selectin. This is in accordance with the ability of Ang-1 to inhibit VEGF induced expression of E-selectin as already reported [45]. The exact reason behind the increased levels of sTie2 in cord serum could be either the shredding of extracellular domain of the Tie2 receptor due to increased proteolytic activity of MMPs [46,47] or due to Golgi mediated release of stored pool of Tie2 [7]. It should be noted that human placental tissue expresses Tie2 receptor and angiopoietins [48,49], with inflammatory mediators and hyperglycemia enhancing its release from the feto-placental endothelial cells and trophoblasts via induction of MMP2, MMP9 and MT1-MMP activities [50-52]. Additionally hyperglycemia is reported to decrease the expression of tissue inhibitor of MMPs i.e. TIMP-2 in trophoblast cells [52]. Although, the exact effect of high levels of sTie-2 in cord blood is presently unknown, it should be noted that it inhibits angiogenesis by limiting the actions of Ang-1 in order to induce vessel destabilization [53]. Soluble Tie2 is also found to reduce angiogenesis in uremic rats [54]. It is hence tempting to speculate that increased sTie2 levels in cord blood serum may also pose danger to the ongoing vasculogenic processes in the developing foetus of GDM subjects. Hence, one can envisage that uncontrolled glycemic status along with inflammatory mediators in placental circulation during GDM will lead to an unopposed release of sTie2, which in turn, may contribute to a leaky feto-placental vasculature thereby enhancing passage of inflammatory mediators from maternal to foetal circulation. This may further trigger endothelial dysfunction in foetal blood vessels to cause future cardiovascular complications. However, more detailed cross-sectional and longitudinal studies are required to confirm this speculation. 

Increased arginase activity in the peripheral blood is reported for type 2 diabetic and breast cancer patients, and its inhibition gives protection from myocardial infarction in rats [55-57]. In normal pregnancy, results are contradictory, with both increased and decreased activity being reported [21,58]. For the current study we observed a significant increase for arginase activity in the cord blood serum of babies born to GDM women. Increased arginase activity in cord blood serum would limit arginine bioavailability and may consequently cause NO deficiency in vascular cells. This could also explain increased eNOS expression in HUVECs obtained from GDM women [36] as a compensatory response to increased arginase activity. Since L-arginine is required to inhibit platelet aggregation and for pregnancy associated immune-tolerance [21,59], depletions in its levels, will also have serious consequences on foetal health. This is exemplified by that fact that enhanced arginase activity contributes to intimal hyperplasia and coronary vascular dysfunction in type2 diabetic animals [60,61]. Initiation of cardiovascular dysfunction occurs in third trimester itself, as reported in a recent study where foetal heart ventricular contractility was reduced for GDM subjects compared to normal pregnancies [38]. These findings suggest that the aberrant foeto-placental environment along with a pro-inflammatory endothelium during second trimester may significantly influence the vascular function of the developing foetus. 

Among the two known isoforms of arginases, the cytosolic arginase (arginase I) is anti-inflammatory [62] while the mitochondrial isoform arginase II, promotes inflammation through mitochondrial reactive oxygen species [63]. Despite our repeated efforts we failed to detect any measurable differences in the mRNA levels of arginase II in HUVECs derived from GDM women, indicating that the likely source for increased arginase activity are the circulating monocytes and macrophages. Intriguingly, accumulation of arginase II expressing macrophages is associated with atherosclerotic lesions [64] and its knock-down prevents macrophage adhesion to endothelium in mice fed high fat diet [22]. Even in ApoE-/- ArgII-/- double knock-out mice, absence of arginase II decreases atherosclerotic lesions [22]. Similarly, in human monocytes, silencing of arginase II decreases their adhesion to endothelial monolayer in addition to reducing production of pro-inflammatory cytokines, TNF-α, IL-6 and MCP-1 by them [22]. Thus, increased arginase activity promotes over-all systemic as well as endothelial inflammation. Although the causative implications of increased arginase activity in GDM and its effect on foetal health are presently unknown, it is tempting to speculate that enhanced arginase activity in feto-placental vasculature may compromise the immune response of the foetus and may predispose the foetus to increased risks of endothelial inflammation and intimal hyperplasia. 

Increased arginase activity in the cord blood in the present study, negatively correlated with glucose intolerance and post load glucose conditions of the mother. It is also worth noting that Arginase II^-/-^ mice have better glucose tolerance and insulin sensitivity [22]. Since glucose intolerance is a co-associated feature of gestational diabetes, increased arginase activity in their cord blood and its association with post-load glucose values may have greater patho-physiological implications which remain to be tested. Whether increased arginase activity is a cause or an effect of glucose intolerance is also unknown though it is reported to be associated with diabetes and endothelial inflammation [55,65]. Another limitation of the current study is the lack of assessment of endothelial function in mothers and the newborns, and absence of information on levels of sTie2 and arginase activity in their peripheral circulation. In conclusion we demonstrate increased inflammation in cord endothelial cells with a concomitant increase in sTie2 levels and arginase activity in cord blood of infants born to Asian Indian GDM women. 

## Supporting Information

Table S1 **Sequences of primers used.**
(DOC)
