# Supplementary material for: Increased Endothelial Inflammation, sTie-2 and Arginase Activity in Umbilical Cords Obtained from Gestational Diabetic Mothers
Source: PLoS One. 2013 Dec 20;8(12):e84546. doi: 10.1371/journal.pone.0084546 (PMC3869849; doi:10.1371/journal.pone.0084546)
Supplement: Table S1 — Sequences of primers used. (DOC) [file pone.0084546.s001.doc]

**Supplement Table 1: Sequences of primers used**

| **Gene Name** | **Sequences** | **Product size (bp)** |
| --- | --- | --- |
| Angiopoietin 2 | 5' GTTGATTTTCAGAGGACTTGG 3' | 453 |
|  | 5' CGAATAGCCTGAGCCTTTCCA 3' |  |
| Arginase II | 5' ATGTCCCTAAGGGGCAGCCTCTCGCGT 3' | 340 |
|  | 5' CACAGCTGTAGCCATCTGACACAGCTC 3' |  |
| E-Selectin | 5' GGCAGTGGACACAGCAAATC 3' | 243 |
|  | 5' TGGACAGCATCGCATCTCA 3' |  |
| VCAM-1 | 5' CGTCTTGGTCAGCCCTTCCT 3' | 454 |
|  | 5' ACATTCATATACTCCCGCATCCTTC 3' |  |
| ICAM-1 | 5' AGGCCACCCCAGAGGACAAC 3' | 406 |
|  | 5' CCCATTATGACTGCGGCTGCTA 3' |  |
| GAPDH | 5' TGCCTCCTGCACCACCAACTGC 3' | 456 |
|  | 5' AATGCCAGCCCCAGCGTCAAAG 3' |  |
